# Multisensory Systems Based on Perfluorosulfonic Acid Membranes Modified with Functionalized CNTs for Determination of Sulfamethoxazole and Trimethoprim in Pharmaceuticals

**DOI:** 10.3390/membranes12111091

**Published:** 2022-11-02

**Authors:** Anna Parshina, Anastasia Yelnikova, Ekaterina Safronova, Tatyana Kolganova, Victoria Kuleshova, Olga Bobreshova, Andrey Yaroslavtsev

**Affiliations:** 1 Department of Analytical Chemistry, Voronezh State University, 394018 Voronezh, Russia; 2 Kurnakov Institute of General and Inorganic Chemistry RAS, 119991 Moscow, Russia

**Keywords:** Donnan potential, potentiometric multisensory system, perfluorosulfonic acid membrane, functionalized CNTs, hybrid materials, ultrasonic treatment, ion transport, sulfamethoxazole, trimethoprim, combination drug

## Abstract

Sulfamethoxazole and trimethoprim are synthetic bacteriostatic drugs. A potentiometric multisensory system for the analysis of sulfamethoxazole and trimethoprim combination drugs was developed. Perfluorosulfonic acid membranes containing functionalized CNTs were used as the sensor materials. The CNTs’ surface was modified by carboxyl, sulfonic acid, or (3-aminopropyl)trimethoxysilanol groups. The influence of the CNT concentration and the properties of their surface, as well as preliminary ultrasonic treatment of the polymer and CNT solution before the casting of hybrid membranes, on their ion-exchange capacity, water uptake, and transport properties was revealed. Cross-sensitivity of the sensors to the analytes was achieved due to ion exchange and hydrophobic interactions with hybrid membranes. An array of cross-sensitive sensors based on the membranes containing 1.0 wt% of CNTs with sulfonic acid or (3-aminopropyl)trimethoxysilanol groups enabled us to provide the simultaneous determination of sulfamethoxazole and trimethoprim in aqueous solutions with a concentration ranging from 1.0 × 10^−5^ to 1.0 × 10^−3^ M (pH 4.53–8.31). The detection limits of sulfamethoxazole and trimethoprim were 3.5 × 10^−7^ and 1.3 × 10^−7^ M. The relative errors of sulfamethoxazole and trimethoprim determination in the combination drug as compared with the content declared by the manufacturer were 4% (at 6% RSD) and 5% (at 7% RSD).

## 1. Introduction

Sulfamethoxazole (SMX) and trimethoprim (TMP) are synthetic bacteriostatic drug substances with a wide range of activity. They are mainly used together in combination drugs. Therefore, there is a necessity to find the methods for their simultaneous determination to improve pharmaceutical quality control and ecological monitoring. Simultaneous determination of SMX and TMP in objects with complex matrixes can be performed by solid-phase extraction with subsequent separation and detection using liquid chromatography–tandem mass spectrometry [1,2,3]. Commercial materials, as well as specially developed materials, for example, molecularly imprinted polymers (MIPs) [4], are applied as extraction sorbents. The use of electrochemical sensors simplifies, to a great extent, the preparation of samples. Composite materials based on graphene, carbon nanotubes (CNTs), and inorganic nanoparticles, which can be introduced in paste [5] or screen-printed electrodes [6,7], as well as deposited on glass carbon electrodes [8], are mainly used in voltammetric sensors for the simultaneous determination of SMX and TMP in natural and tap waters, as well as in urine and serum (Table 1). The analysis of SMX and TMP combination drugs was performed with glass carbon [9] and diamond electrodes dopped with boron [10] (Table 1). For these sensors, a special pretreatment of the materials before every measurement or after a small series of measurements was required for a reliable analysis [5,6,8,9]. The number of works devoted to the development of potentiometric sensors for the individual [11,12,13,14,15,16] and simultaneous (Table 1, [17]) determination of SMX and TMP was not too large. Most studies suggested the utilization of ionophores based on MIPs [12,14,15,16,17]. However, the expected high selectivity of the interactions between MIP and analyte decreases in the presence of related analytes, as well as due to the nonspecific interactions of the polymer matrix with the components of the analysis object. Partial extraction of the bound analyte and a difference in the constants of repeated interaction also decrease selectivity [18].

A low selectivity of single sensors can be compensated by the use of a multisensory approach, in which responses from an array of sensors with cross-sensitivity are measured in multicomponent solutions and processed using special chemometric methods [19,20,21]. At the same time, a response from a single sensor does not provide the full information about the object of analysis. Traditional membranes with ionophores [19,20,21], as well as novel hybrid materials [22,23,24,25], can be used as materials for potentiometric multisensory systems. The use of the Nafion-type perfluorosulfonic acid membranes in cross-sensitive sensors with the Donnan potential (DP) analytical signal was described in [23,24,25]. The Nafion-type membranes have a system of hydrophilic pores and channels, which are of the same size in the hydrated state as organic analyte ions. The microstructure and conducting properties of such membranes can be affected by modification or physical treatment [26]. This changes conditions for ion exchange and sorption of organic and inorganic ions. The high mechanical and chemical stability of perfluorosulfonic acid membranes provides their low proneness to fouling and fast and effective regeneration [27]. Hybrid materials based on the Nafion membrane and its Russian analog, the MF-4SC membrane, containing CNTs, poly(3,4-ethylenedioxythiophene), and polyaniline, were used for the simultaneous determination of sulfacetamide and inorganic components in pharmaceuticals [24], as well as simultaneous determination of sulfacetamide and sulfanilamide in the UV-degraded pharmaceuticals [25]. The introduction of π-conjugated moieties and proton-acceptor groups into the perfluorosulfonic acid membranes resulted in the improvement of the sensor sensitivity to 4-aminobenzenesulfonic acid derivatives due to an increase in the number of sorption centers and changes in the size of the intrapore space.

CNTs are of great interest for electrochemical sensors because of their good electron conductivity, large surface area, satisfactory mechanical and chemical stability, as well as the possibility of modifying their surface by the formation of covalent bonds or adsorption [28,29,30]. Surface modification of CNTs notably extends their functions in the composition of the sensors. In voltammetric sensors, apart from the function of an electron signal transducer, CNTs are used for binding reactive centers (4-aminobenzoic acid [31], β-cyclodextrin [32], ferrocene derivatives with β-cyclodextrin [33], chitosan with nickel ions [34], and MIPs [35,36]). For example, MIP synthesis on the CNT surface improves the kinetics and effectiveness of the binding of analytes [35,36]. CNTs are widely used as electron conductors in wearable potentiometric sensors with a solid contact [37,38]. The problem of ionophores leaching from the membranes of potentiometric sensors can be solved by their binding on the CNT surface [39]. The reviews [28,40] discuss the achievements and prospects in the field of CNTs’ use in biosensors with the electrochemical type of detection. Different approaches to enzyme and antibody immobilization on the surface of CNTs provide their application to such analytical devices.

Hybrid materials based on CNTs and Nafion-type membranes are of great interest for use in different applications as materials with mixed electron-ionic conductivity. They are primarily studied for utilization in membrane-electrode assemblies of hydrogen-air fuel cells [41]. Functionalization of the CNT surface enables decreasing their aggregation and varying their distribution within hydrophilic and hydrophobic membrane phases. Incorporation into the Nafion-type membranes of dopants with different natures gives us an opportunity to change their properties [26,41,42,43]. The presence of dopants with acidic groups on the surface results in an increase in water uptake and ionic conductivity due to the additional current carriers. At the same time, the presence of dopants with proton-acceptor properties results in their interaction with sulfonic acid groups of the membrane and a change in water balance, ionic conductivity, and selectivity of the membranes. It was shown that the incorporation of CNTs with proton-donor (-SO_3_^−^) and proton-acceptor (-NH_x_^+^) groups resulted in the changing of the sorption and transport properties of membranes [44,45]. The works [24,46] showed that potentiometric sensitivity to the ions of drug substances with aromatic moieties could be improved due to the introduction of CNTs, preliminarily treated with an oxidant, into MF-4SC membranes. The formation of carboxyl groups on the CNT surface favored their partial localization in the membrane’s hydrophilic pores. The presence of CNTs within the pores provided the centers for stacking interactions with the analytes. At the same time, the localization of CNTs in the hydrophobic matrix affected the flexibility of the membrane and, as a result, influenced the sorption ability of bulk organic ions. Modification of CNTs with sulfonic acid and amino-containing groups should facilitate an increase in their affinity for SMX and TMP since they have in their structure both acidic and basic groups. Nafion-type membranes containing CNTs with proton-donor and proton-acceptor functional groups have not been studied for use as materials of potentiometric sensors. At the same time, an increase in the sorption ability of a material due to ion exchange and hydrophobic interactions with an analyte and a change in the membrane system of pores and channels may be interesting.

The aim of this work was the development of a potentiometric multisensory system based on the perfluorosulfonic acid membranes containing CNTs with the surface functionalized by groups with different acid-base properties for the analysis of SMX and TMP combination drugs.

## 2. Materials and Methods

### 2.1. Materials and Reagents

A dimethylformamide solution of the MF-4SC perfluorosulfonic acid polymer in the lithium form (10 wt%, equivalent weight is 1100; Plastpolymer, Saint-Petersburg, Russia); Taunit S12 multiwall CNTs (outer diameter is 20–40 nm, inner diameter is 5–10 nm, obtained by CVD catalytic pyrolysis of hydrocarbons on Ni/Mg catalyst; NanoTechCenter, Tambov, Russia); nitric acid (special purity, >70% HNO_3_, Chimmed, Moscow, Russia); *p*-toluenesulfonic acid (97.5%, Acros Organics, Geel, Belgium); *D*-glucose (Ph Eur, hydrated form, Merck, Darmstadt, Germany); ethanol (95%, Ferein, Minsk, Belarus); (3-aminopropyl)trimethoxysilane (97%, Alfa Aesar, Ward Hill, MA, USA); acetone (reagent grade, >99.75%, Chimmed, Moscow, Russia); hydrochloric acid (special purity, 35–38%, Chimmed, Moscow, Russia); potassium chloride (reagent grade, Chimmed, Moscow, Russia); sulfamethoxazole (4-amino-*N*-(5-methyl-3-isoxazolyl)benzenesulfonamide, 98%, Alfa Aesar, Ward Hill, MA, USA), trimethoprim (2,4-Diamino-5-(3,4,5-trimethoxybenzyl)pyrimidine, ≥99%, Alfa Aesar, Ward Hill, MA, USA); “Biseptol^®^ 120 mg” tablets (Adamed Pharma, Czosnów, Poland); deionized water (resistance 18.2 MΩ, pH 5.41 ± 0.05) were used.

### 2.2. Preparation of Model Solutions

**Figure 1:**
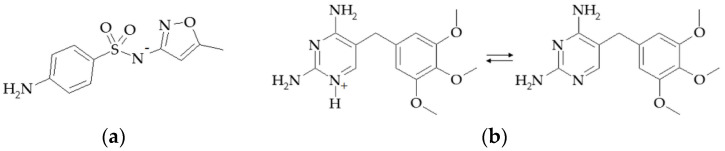
The structure of SMX^−^ (**a**) and TMP^+^/TMP (**b**).

Model solutions of SMX and TMP at various concentration ratios ranging from 1.0 × 10^−5^ to 1.0 × 10^−3^ M were prepared for potentiometric measurements. The pH values of the solutions depended on the concentrations of drug substances and ranged from 4.53 to 8.31. Additional reagents for pH correction were not used. Under these conditions, SMX was in the anionic form (SMX^−^), and TMP was in the cationic and molecular forms (further, cations and molecules of TMP, which were in equilibrium, were designated as TMP^+^/TMP) (Figure 1).

Calibration solutions of SMX and TMP with various concentration ratios ranging from 6.0 × 10^−6^ to 6.0 × 10^−5^ M were prepared for spectrophotometric analysis. An ammonium buffer solution (pH 10.0) was used to set the pH of the solutions.

### 2.3. Preparation of Pharmaceutical Solutions

The composition of one Biseptol^®^ 120 mg tablet was SMX (100.0 mg), TMP (20.0 mg), potato starch (44.25 mg), talk powder (3.75 mg), magnesium stearate (1.25 mg), and polyvinyl alcohol (0.75 mg).

The pharmaceutical stock solution with a volume of 1 L was prepared by the dissolution of 1 tablet in deionized water. SMX and TMP concentrations in the stock solution were 3.948 × 10^−4^ and 6.889 × 10^−5^ M, respectively. This solution was filtrated before sampling for analysis. The pharmaceutical stock solution was diluted 10 times for potentiometric and spectrophotometric analysis.

### 2.4. Functionalization of CNTs

**Figure 2:**
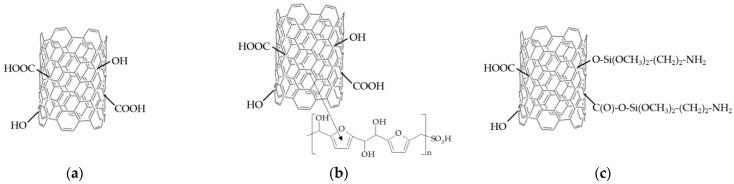
The scheme of the surface fragments of functionalized CNTs: CNTs-COO^−^ (**a**), CNTs-SO_3_^−^ (**b**), and CNTs-NH_3_^+^ (**c**).

CNTs were functionalized by the treatment of commercial CNTs according to the methods described elsewhere [47,48]. Primarily, CNTs were cleaned from the remains of the catalyst used in their synthesis with simultaneous oxidation. For this purpose, a CNT suspension in a 30% HNO_3_ solution in a 1/8 weight ratio was prepared and kept for 1 h at 90 °C under constant stirring. Then they were washed with a large amount of water to a neutral pH and dried in air at 90 °C for 24 h. Such treatment provides the formation of carboxyl and hydroxyl groups on the CNT surface due to some C-C bonds breaking with subsequent oxidation (Figure 2a).

The sulfonation of the cleaned CNTs was conducted under hydrothermal conditions in the presence of *p*-toluenesulfonic acid and *D*-glucose (in the ratio of 0.2 g of CNTs, 0.25 g of *p*-toluenesulfonic acid, 0.25 g of *D*-glucose, 20 mL of water) at 180 °C for 24 h. *D*-glucose has been used as the carbon source for the attachment of -SO_3_H groups [49]. Under synthesis conditions, *D*-glucose was dehydrated, then hydrothermally carbonized into a polymer that reacted with *p*-toluenesulfonic acid. The product was bound with the CNT surface by stacking interactions (Figure 2b). The obtained mixture was centrifuged and repeatedly washed with deionized water to a neutral pH. After that, it was additionally washed with ethanol and dried at 110 °C for 24 h.

The cleaned CNTs were treated with a 6 M HNO_3_ solution at 90 °C for 1 h to increase the number of carboxylic groups on the surface. Then they were washed with a large amount of water to neutral pH and dried in the air at 90 °C for 24 h. The product was usedto obtain CNTs with amine-containing groups on their surface. A 5 wt% solution of (3-aminopropyl)trimethoxysilane in acetone was added to the water dispersion with 1 wt% of the obtained CNTs (to prepare a mixture with 1/10 weight ratio of (3-aminopropyl)trimethoxysilane and CNTs). Then the mixture was kept at 80 °C with constant stirring for 30 min, washed with water, and dried. As a result, -O-Si(OCH_3_)_2_-(CH_2_)_3_-NH_3_^+^ groups were formed on the CNTs surface (Figure 2c).

Thus, CNTs with a surface covalently modified by carboxyl and (3-aminopropyl)trimethoxysilanol groups, as well as non-covalently by sulfonic acid groups, were prepared. Further, the functionalized CNTs were designated as CNTs-COO^−^, CNTs-NH_3_^+^, CNTs-SO_3_^−^, or CNTs-X in general.

### 2.5. Membrane Preparation

**Figure 3:**
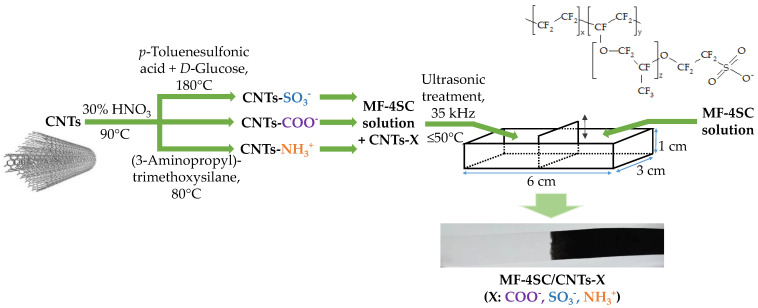
The scheme of preparation of the MF-4SC/CNTs-X hybrid membranes. A membrane with gradient distribution of CNTs along the length is shown.

Hybrid materials based on the MF-4SC perfluorosulfonic acid membrane and functionalized CNTs were manufactured. Uniformly modified membranes were obtained for the investigation of the equilibrium and transport properties. Membranes with gradient modifications along their lengths were obtained for use as sensing materials; only half of the film contained the dopant. Further, the membranes with functionalized CNTs were designated as MF-4SC/CNTs-COO^−^, MF-4SC/CNTs-NH_3_^+^, MF-4SC/CNTs-SO_3_^−^, or MF-4SC/CNTs-X in general. The scheme of preparation of the hybrid membrane is shown in Figure 3.

Hybrid membranes were manufactured by a casting procedure. For this purpose, previously prepared CNTs-X in an amount, which was necessary to obtain membranes with 0.5, 1.0, or 1.5 wt% of the dopant, were added to an MF-4SC polymer solution. After that, the mixture was treated with an RK-100 ultrasonic (US) cleaner sonication bath (frequency—35 kHz, Bandelin electronic, Berlin, Germany) filled with water. The mixture of the polymer solution with the dopant was placed into a glass hermetically closing vessel and immersed in the US bath. The level of liquid in the vessel and the level of water in the US bath were the same. The bottom of the glass vessel was 0.5 cm higher than that of the US bath. US treatment was performed for 45 min at a temperature not greater than 50 °C. The obtained suspension was cast on a glass surface. Films were formed in rectangular cells (the cell length, width, and height were 6, 3, and 1 cm, respectively) with a moving part in the center to manufacture gradient-modified materials. A polymer solution without the dopant and US treatment from one end and the suspension of the dopant in the polymer solution treated by the US from the other were simultaneously cast (Figure 3). The solvent was removed by drying in a vacuum drying oven (Jeio Tech, Daejion, Korea) at 60 °C (4 h), 80 °C (12 h), and 110 °C (4 h). The obtained films were hot-pressed under a pressure of 5 MPa at 110 °C for 3 min to provide better endurance.

Thus, the prepared hybrid membranes were bulk-modified films. The gradient-modified membrane was a phase, just one part of which contained another phase, namely CNTs. The gradient-modified membrane contained a dopant up to half of its length. The conditions of hybrid membrane preparation were chosen in a way that the dopant distribution in the modified part was uniform. However, there was an intermediate part between modified and unmodified parts. It was formed as a result of mixing the solutions of the polymer without the dopant and the polymer with the dopant upon membrane casting. The intermediate part did not introduce an error in the DP sensor response since the membrane ends were immersed in the test and reference solutions only to 0.3–0.5 cm and the length of the membrane was 6 cm. The measurement time of DPsensors response was less than 1 min. It was enough to establish a quasi-equilibrium near the interface of the membrane and the solutions, but the composition of the other membrane part did not change.

Two MF-4SC membranes were manufactured as reference samples. One of them was cast from an initial polymer solution, and another from a polymer solution treated by the US (further, this membrane was designated as MF-4SC-US).

The density of the prepared membranes in a dry state was 1.5–1.6 g/cm^3^, and their thickness was 155 ± 10 μm. The thickness of the MF-4SC, MF-4SC-US, MF-4SC/CNTs-COO^−^, MF-4SC/CNTs-SO_3_^−^, and MF-4SC/CNTs-NH_3_^+^ membranes in a swollen state was 232 ± 10, 228 ± 4, 195 ± 6, 192 ± 14, and 273 ± 16 μm, respectively. The membrane image is shown in Figure 3.

All the prepared membranes were conditioned to standardize and convert into the H^+^-form. For this purpose, they were double-treated with a 5% HCl solution at room temperature for 1.5 h and then washed with deionized water until a negative reaction for Cl^−^ ions occurred. To convert membranes into the K^+^-form, they were kept in a 2 M KCl solution for 72 h, followed by washing them in deionized water. The membranes were regenerated in the same way after long-term use (up to 3 months). After a series of repeated measurements (~100 times), the membranes were first kept in a 0.1 M KCl solution for 30 min under constant stirring and then placed for storage in deionized water.

### 2.6. Apparatus and Experiment Procedure

The density of the polymer solutions was determined before and after US treatment using a portable densimeter Densito (Mettler Toledo, Greifensee, Switzerland), at 25 ± 0.1 °C. The viscosity of the polymer solutions was evaluated immediately after the treatment with an oscillatory viscosimeter SV-1A (A&D, Tokyo, Japan) at 25 ± 0.1 °C. The dynamic viscosity (η, mPa∙s) was calculated as the ratio of viscosity to solution density. The calibration was performed using 5 mPa∙s and 10 mPa∙s viscosity standards (Brookfield, Toronto, ON, Canada).

The IR–spectra of the CNTs and membranes in a dry state were recorded on a Nicolet iS5 FTIR spectrometer (Thermo Scientific, Waltham, MA, USA) with the Fourier transformation and ATR add-on (a diamond crystal). The microstructure and the chemical composition of CNT samples were analyzed using a Carl Zeiss NVision 40 scanning electron microscope (with an accelerating voltage of 1 kV).

The ion-exchange capacity (IEC, mmol/g) of CNTs and membranes in hydrogen form was determined by acid-base titration. The CNTs-X powder weighing 0.1–0.2 g was placed in 10 mL of 0.5 M NaCl (in the cases of CNTs-COO^−^ and CNTs-SO_3_^−^) or 0.1 M HCl (in the case of CNTs-NH_3_^+^). Then it was constantly stirred for 4 h. The obtained suspension was titrated with 0.05 M NaOH solution. A dry membrane weighing 0.1–0.2 g was placed in 50 mL of 0.1 M NaCl for 4 h. The salt solution was subsequently removed and titrated with a 0.05 M NaOH solution. The IEC was calculated per weight of the dry membrane, which was determined after keeping the membrane at 150 °C for 30 min.

The water uptake of membranes was determined as the weight difference of the membrane in a swollen (after long-term contact with water) and dry state. The membrane thermal treatment was conducted in platinum crucibles in an argon atmosphere at temperatures varying from 20 to 150 °C using a Netzsch-TG 209 F1 thermal balance (Netzsch, Selb, Germany).

The ionic and electronic conductivities of the membranes were determined at temperatures varying from 25 to 60 °C in their contact with deionized water. The measurements were performed using an Elins Z500 PRO impedance meter (Elins, Chernogolovka, Russia) in the frequency range from 10 Hz to3 MHz in a potentiostatic mode with an amplitude of 80 mV. The measurements were performed using graphite paper, membrane, and graphite paper symmetric cells with an electrode-active surface area of ~1 cm^2^. The electronic conductivity was calculated from the resistance to the direct current. The electronic conductivity of all samples was negligibly small (less than 1% of total conductivity). The ionic conductivity (σ, S/cm) was calculated from the resistance. The resistance value was obtained as the cutoff along the axis of active resistance in the impedance spectrum.

A membrane was placed in a cell between two chambers to estimate the diffusion permeability; the volume of each one was 32 cm^3^. The first chamber was filled with 0.5 M KCl solution, and the second one with 0.02 M KCl solution. The solutions were continuously stirred using magnetic stirrers. During experimental runs, the conductivity was measured using an Expert-002 conductometer (Ekonix-Expert, Moscow, Russia) in the chamber, which initially contained a 0.02 M KCl solution. The experiments were performed at ~25 °C. The diffusion permeability of the membranes was calculated as follows:

(1) $$P=\frac{dc}{dt}\cdot \frac{V\cdot l}{S\cdot \Delta c}\;,$$

where *V* is the volume of the solution, cm^3^; *l* is the membrane thickness, cm; ∆*c* is the concentration gradient, mol/cm^3^; *t* is time, s; S is an active surface of the membrane (4.9 cm^2^).

Spectrophotometric analysis of the pharmaceuticals containing SMX and TMP was performed using a Shimadzu UV-1800 spectrometer (Shimadzu, Kyoto, Japan).

The cell scheme for the response evaluation of systems of DP-sensors based on membranes with different compositions was thoroughly described elsewhere [23,24]. The membranes 6 cm in length and 0.5 cm in width in the K^+^-form were used in DP-sensors. Membranes connected reference and test solutions as bridges. The distance between the membrane interface with the test solution and the reference solution corresponded to the membrane length. It reduced the influence of diffusion and migration on the DP-sensor response. The modified end of the membrane was immersed in the central section with the test solution using a multi-section cell, and the other (unmodified) end of the membrane was immersed in separate sections with the reference solutions. The dopant absence provided the composition closeness of the reference solution and the intrapore solution, leveling the potential difference at the corresponding interface. The ESr-10103 silver chloride electrodes and the ES-10301/4 glass electrode (Econix-Expert, Moscow, Russia) were used. A potential difference was measured using a multichannel potentiometer between a silver chloride electrode (connected to the reference electrode input) immersed in the test solution and silver chloride electrodes (connected to the measurement inputs) immersed in the reference solutions. The pH of the test solution was simultaneously measured. The voltages of several circuits (Equation (2)) for the membranes with different compositions were measured.

Ag | AgCl, sat. KCl | 1M KCl | membrane | test solution | sat. KCl, AgCl | Ag(2)

Chronopotentiometry was used to evaluate the response time and stability of the DP-sensors. The response of the DP-sensors based on the unmodified and hybrid membranes, which were in contact with a solution containing SMX and TMP for 1 h, reached its constant value for less than 1 min. The further changes (the response drift) were comparable with the value scattering when repeating the experiment. The reproducibility variance values of the DP-sensors response were from 13 to 26 mV^2^.

### 2.7. Data Processing Procedure

Multidimensional regression analysis was used to estimate systems of calibration equations when performing spectrophotometric and potentiometric measurements. For this purpose, analytical signals were evaluated in solutions containing both drug substances. The factors values (p*c* in the case of potentiometry and *c* (M) in the case of spectrophotometry) were varied in constant and equal steps to eliminate correlation and provide good conditioning of multidimensional regression equations. The algorithm for experiment planning, as well as the algorithm for calculating the equation coefficients using the least square method, were thoroughly described elsewhere [25]. The significance of the calibration equations coefficients was evaluated by Student’s *t*-test. The variance of reproducibility and the variance of adequacy by Fisher’s *F*-criterion were compared with the control adequacy of the calibration equation.

Calibration equations in the form of Equation (3), which took into account the influence of the DP-sensor response (∆φ*_D_*, mV) on the negative decimal logarithm of the concentration of SMX^−^ anions (*pSMX*), the negative decimal logarithm of the overall concentration of TMP molecules and TMP^+^ cations (*pTMP*) and pH, were established for different compositions of DP-sensors:

(3) $$\Delta \varphi_{D}=b_{0}+b_{1}\cdot pSMX+b_{2}\cdot pTMP+b_{3}\cdot pH,$$

where *b_0_* is the constant term of the calibration equation, and mV; *b_i_* is the sensitivity coefficient of the DP-sensor to the *i*-th component, mV/p*c*.

The calibration characteristics of the DP-sensors were re-established after a year of use to prove their stability. The values of the calibration equation coefficients and their variances were compared with a Student’s *t*-test and Fisher’s *F*-criterion, respectively.

DP-sensors with a minimal correlation between their responses according to the *r*-criterion were chosen for multisensory systems (the number of sensors in an array was the same as the number of analytes). The system of calibration equations was solved by a matrix approach to calculate the analyte concentrations in the analysis object. Limits of detection (LODs) of the analytes for the system of calibration equations were estimated by the 3σ rule.

Calibration equations for spectrophotometric analysis were established at pH = 10.0 at two wavelengths (257 and 287 nm). Calibration equations as in Equation (4), which took into account the influence on the absorbance (A) of the molar concentrations of SMX (*c_SMX_*, M) and TMP (*c_TMP_*, M), were established for both wavelengths:

(4) $$A=a_{1}\cdot c_{SMX}+a_{2}\cdot c_{TMP},$$

where *a_i_* is the sensitivity coefficient to the *i*-th component, M^−1^.

The results of the potentiometric analysis of the preparation were compared with its composition as declared by the manufacturer and with the results of the spectrophotometric analysis to evaluate the accuracy.

## 3. Results and Discussion

### 3.1. Properties of CNTs

**Figure 4:**
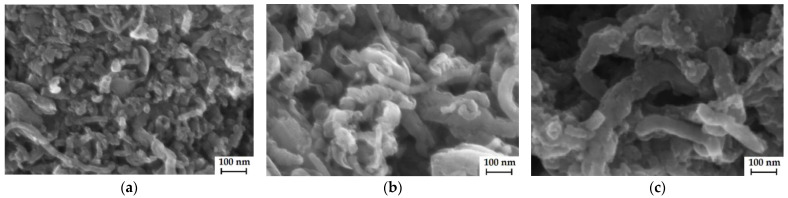
SEM micrographs of the CNTs-COO^−^ (**a**), CNTs-SO_3_^−^ (**b**), and CNTs-NH_3_^+^ (**c**).

According to the SEM micrographs (Figure 4), the average diameter of all types of CNTs is 20–40 nm. The functionalization of CNTs was proven by changes in the IEC and FTIR spectra. The data were discussed in our previous works in detail [44,45].

The IEC values of the dopants were 0.014, 0.27, and 0.64 mmol/g for CNTs-COO^−^, CNTs-SO_3_^−^, and CNTs-NH_3_^+^, respectively. The higher IEC value of CNTs-NH_3_^+^ was due to the additional stage of CNT carboxylation and different mechanisms of interaction.

**Figure 5:**
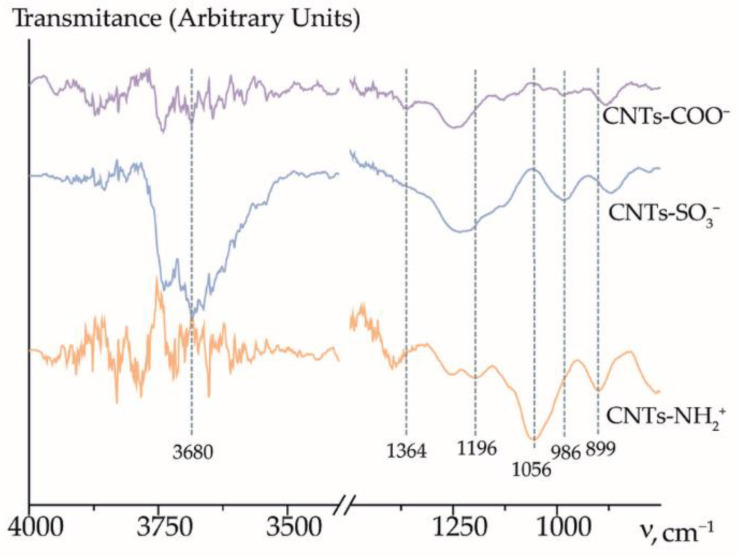
FTIR spectra of CNTs-COO^−^, CNTs-SO_3_^−^, and CNTs-NH_3_^+^.

The FTIR analysis of all the types of CNTs confirmed the functionalization (Figure 5).

The weak absorption band at 1364 cm^−1^ was observed in the spectrum of CNTs-COO^−^ due to the O-H vibrations of the carboxyl group. The absorption bands at 986 and 1178 cm^−1^ were observed in the spectrum of CNTs-SO_3_^−^. These bands corresponded to the vibrations of sulfonic acid groups. The functionalization of CNTs (3-aminopropyl)trimethoxysilanol groups resulted in the appearance of the bands at 1056 and 899 cm^−1^ corresponding to the vibrations of primary amines and 1196 cm^−1^ corresponding to the vibrations of Si-O groups. Absorption bands in the O-H stretching region (3700–3500 cm^−1^) were observed. The sulfonation of CNTs led to a substantial increase in the intensity and width of the stretching O-H bands at 3700–3500 cm^−1^, which was caused by the appearance of both the -SO_3_H groups and a considerable amount of water molecules upon the hydration of these acid sites. According to the TGA analysis with mass spectroscopy of the gases evolved, functionalizing groups on the CNTs surface are thermally stable up to the temperature >200 °C [44,45]. These data support the efficiency of CNTs functionalization by -SO_3_^−^ and -O-Si(OCH_3_)_2_-(CH_2_)_3_-NH_3_^+^ groups. The functionalized CNTs were quite suitable as dopants for MF-4SC-type membranes with an operating temperature of <120 °C.

### 3.2. Properties of Membranes

The structure of perfluorosulfonic acid membranes was formed by the hydrophobic matrix and hydrophilic pores connecting to each other with channels. Fixed sulfonic acid groups were located along the inner surface of the pores. The influence of the film formation conditions and the solution composition on the microstructure of the prepared membranes and the CNT distribution between the hydrophobic matrix and hydrophilic pores and channels could be estimated from the analysis of the equilibrium and transport properties of the membranes.

**Figure 6:**
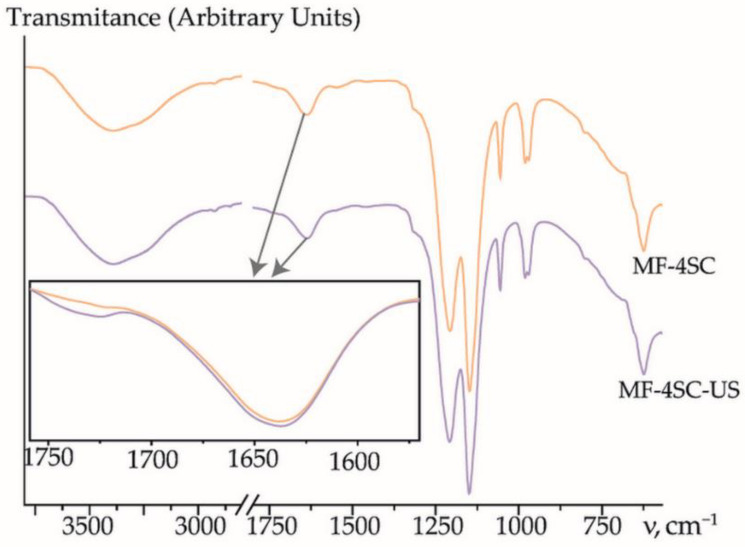
FTIR spectra of MF-4SC and MF-4SC-US membranes in a dry state.

US treatment of the MF-4SC polymer solution leads to a decrease in its viscosity from η = 82.5 ± 0.4 mPa·s for the pristine solution to η = 64.6 ± 0.6 mPa·s for the MF-4SC solution after US treatment for 45 min without the dopant, and to 58–62 mPa·s for the solutions containing the dopant after US treatment. According to [50], this was caused by deagglomeration as well as by a decrease in the average molecular weight of the polymer. The US treatment caused the break of macromolecules that occurred close to the centers. This was due to the difference in the rates of motion of solvent molecules and polymer macromolecules upon rarefaction and compression. A peak with low intensity at 1740–1745 cm^−1^ appeared on the FTIR spectra of the membranes prepared from the solutions after US treatment. It was referred to as the carboxyl group formation (Figure 6).

**Figure 7:**
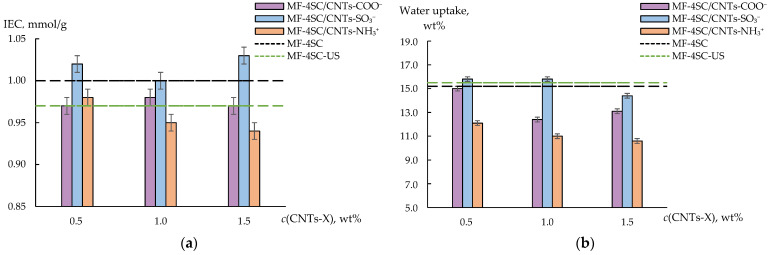
IEC (mmol/g) of the membranes (**a**) and water uptake (wt%) of the membranes in the K^+^-form in contact with water (**b**). The confidence intervals in the evaluation of the IEC and water uptake were ± 0.01 mmol/g and ± 0.2%, respectively, in the case of MF-4SC membrane, and ± 0.01 mmol/g and ± 0.1%, respectively, in the case of MF-4SC-US membrane.

The IEC of the MF-4SC membrane decreased due to the US treatment of the solution from 1.00 to 0.97 mmol/g (Figure 7a). This was caused by the loss of a part of the sulfonic acid groups under the US treatment due to breaks in the polymer chains. CNTs have hydrophobic nature; hence they should be primarily located in the membrane hydrophobic matrix, but the presence of hydrophilic groups on the surface of CNTs-X promotes their partial localization in the membrane hydrophilic pores. The IEC values of MF-4SC membranes, containing CNTs-COO^−^ or 0.5 wt% CNTs-NH_3_^+^, do not differ from that of MF-4SC-US membrane if the errors of the IEC are taken into account (Figure 7a). Sulfonic acid groups on the surface of CNTs, which are present in pores, increase the membrane IEC up to 1.00–1.03 mmol/g (Figure 7a). The IEC of MF-4SC/CNTs-NH_3_^+^ membranes decreased to 0.94–0.96 mmol/g at the dopant concentration of 1.0–1.5 wt% (Figure 7a) due to the bonding of sulfonic acid groups to the membrane. At the same time, not all amino-containing groups of CNTs present in pores may be available for the formation of bonds with sulfonic acid groups of the membrane. Therefore, the difference between the IEC of hybrid membranes and MF-4SC-US was more pronounced upon modification by CNTs-SO_3_^−^, despite the fact that the IEC of CNTs-NH_3_^+^ is two times more than the CNTs-SO_3_^−^. Besides, the differences in the hydrophilicity of the surface and the affinity of hydrophilic groups in the dopant and polymer should have a great influence on the process of the system of pores and channels formation and, as a result, on the change in the IEC.

The water uptake of the membrane prepared from the polymer solution after the US treatment is close to the water uptake of the pristine MF-4SC membrane (Figure 7b). A possibility for the sorption of the same amount of water molecules by MF-4SC and MF-4SC-US membranes despite a decrease in the IEC of the latter may be due to deagglomeration of macromolecules and an increase in the mobility of single units upon the formation of the membrane from the US treated solution. This led to the formation of a system of pores and channels with better connectivity. MF-4SC/CNTs-COO^−^ and MF-4SC/CNTs-NH_3_^+^ membranes have lower water uptake than MF-4SC and MF-4SC-US membranes (Figure 7b). It should be noted that a decrease in the water uptake with an increase in the concentration of the dopant for MF-4SC/CNTs-NH_3_^+^ membranes was more pronounced (Figure 7b). When the concentration of CNTs-SO_3_^−^ was 0.5–1.0 wt%, the membrane’s water uptake was slightly higher (15.8 wt%) than that of the unmodified membranes. When the concentration of CNTs-SO_3_^−^ was 1.5 wt%, the water uptake decreased to 14.4 wt% (Figure 7b). CNTs-COO^−^ prevented pores from stretching during membrane hydration since they were mainly located in the perfluorinated matrix owing to the small number of functional groups on the surface. CNTs-SO_3_^−^ favor water sorption due to the increasing sulfonic acid group concentration in the membrane pores. CNTs modified with (3-aminopropyl)trimethoxysilanol groups displaced water from the membrane pores because of steric factors and the formation of strong bonds between amine-containing moieties and sulfonic acid groups.

The US treatment of the polymer solution led to a slight increase in the membrane ionic conductivity and a great increase in the diffusion permeability (Table 2). The more developed and connected system of pores and channels, owing to macromolecule deagglomeration and the increase in unit mobility, facilitates the transport of both counter-ions along the walls of the pores and channels and co-ions in their bulk. A less pronounced rise in the ionic conductivity compared with the diffusion permeability was due to a decrease in the number of charge carriers because of a loss of some SO_3_^−^ groups when separating the polymer sidechains under US.

The conductivity of hybrid membranes is higher as compared with the MF-4SC membrane (Table 2) due to the better connectivity of the pores and channels caused by the US treatment of the polymer solution. At the same time, the ionic conductivity of MF-4SC/CNTs-COO^−^ and MF-4SC/CNTs-NH_3_^+^ membranes was lower than that of MF-4SC-US membrane (Table 2). This was due to the lowering of the IEC and water uptake of these membranes compared with those of the MF-4SC-US membrane (Figure 7). The dependence of the ionic conductivity on CNTs-COO^−^ and CNTs-NH_3_^+^ content passed through the maximum at 1.0 wt%. A decrease in the conductivity at 1.5 wt% of the dopant was caused by the strong decrease in the water uptake of the membranes (Figure 7b). At the same time, the ionic conductivity of MF-4SC/CNTs-SO_3_^−^ membranes monotonically increased with the growth of the dopant concentration. When the concentration of CNTs-SO_3_^−^ was 1.5 wt%, it reached higher values than for the MF-4SC-US membrane (Table 2). This was due to the formation of additional charge carriers owing to the increasing concentration of cation-exchange groups in the pores.

The diffusion permeability of MF-4SC/CNTs-COO^−^ membranes was close to that of the MF-4SC-US membrane and slightly exceeded it at 1.0 wt% of CNTs-COO^−^ (Table 2). The diffusion permeability of the MF-4SC/CNTs-SO_3_^−^ membranes at the dopant concentration equal to 0.5–1.0 wt% was lower than that of MF-4SC-US and MF-4SC/CNTs-COO^−^ membranes. The increase in the CNTs-SO_3_^−^ concentration within the membrane from 0.5 to 1.5 wt% led to the monotonic increase in diffusion permeability (Table 2). A decrease in the diffusion permeability of the membranes with CNTs-SO_3_^−^ concentration of 0.5–1.0 wt% as compared with the MF-4SC-US membrane despite enhanced water uptake was due to the increasing concentration of sulfonic acid groups preventing the anion transfer into the pores. At the same time, the diffusion permeability of the membrane with 1.5 wt% of CNTs-SO_3_^−^ increases. Probably, upon membrane casting in the presence of a high concentration of CNTs with hydrophilic groups, large pores or caverns may be formed. The rate of anion transport through such caverns is high, but their presence does not affect the rate of cation transport. A similar effect was described in [51]. The presence of extended and rigid dopants, such as CNTs, inside or near the pores limits the mobility of macromolecule units and makes it difficult to rearrange the membrane microstructure. The diffusion permeability of MF-4SC/CNTs-NH_3_^+^ membranes is significantly higher than that of the other modified and unmodified membranes (28.5×10^−8^–56.5×10^−8^ cm^2^/s) and reaches its maximum at 1.0 wt% (Table 2). The decrease in the concentration of sulfonic acid groups and the formation of anion-exchange centers facilitates nonselective transport through the membrane. It is possible that the simultaneous presence of the polymer with sulfonic acid groups and the CNTs with bulk -O-Si(OCH_3_)_2_-(CH_2_)_3_-NH_3_^+^ groups affected the self-organization of hydrophilic clusters around the dopant and the distribution of the latter between hydrophobic and hydrophilic phases when casting the membranes. This could lead to a larger size of pores and connecting channels and a higher dopant concentration in the pores. This was also facilitated due to a higher concentration of hydrophilic groups on the CNTs-NH_3_^+^ surface compared with other functionalized CNTs. At the same time, nonmonotonic changes in the diffusion permeability may be caused by different availability of bulk surface groups for interactions.

### 3.3. Characteristics of the DP-Sensors

#### 3.3.1. Cross-Sensitivity of the DP-Sensors

**Figure 8:**
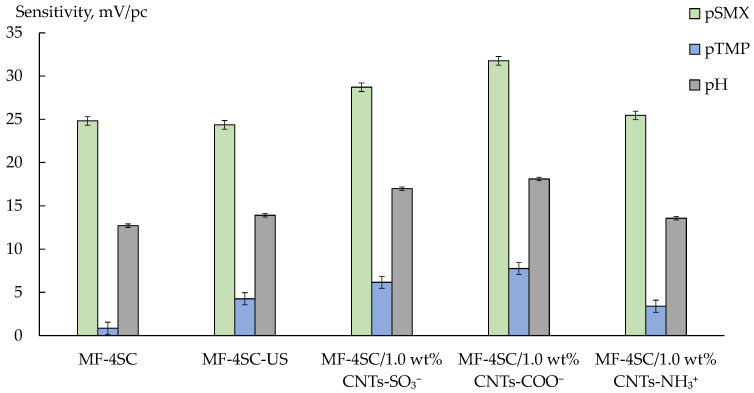
Sensitivity coefficients of DP-sensors based on MF-4S/CNTs membranes to SMX^−^, TMP^+^/TMP, and water dissociation products (1.0 × 10^−5^–1.0 × 10^−3^ M, pH 4.53–8.31).

DP-sensors, based on the studied membranes, including the unmodified ones, had a sufficiently high sensitivity to SMX^–^ anions and water dissociation products. At the same time, the sensitivity to TMP, which was in the cationic and partially molecular forms, was low (Figure 8).

The pH value of an intrapore solution within the cation exchange membrane was lower than that of an inner solution by ~2 units owing to the Donnan exclusion of OH^−^ ions [52]. Therefore, the molecular form of TMP in the membrane should be turned into the cationic one due to the protonation of nitrogen in the pyrimidine ring. At the same time, TMP molecules are characterized by high hydrophobicity. It limits their sorption into hydrophilic membrane pores. This is the reason for the low sensitivity of DP-sensors to TMP^+^/TMP despite the cation-exchange nature of the MF-4SC membrane.

At the same time, the MF-4SC membrane was not absolutely selective, so the transfer of organic anions into the membrane was possible. Meanwhile, the state of SMX^−^ anions in the membrane phase and inner solution should differ due to the analyte ampholytic properties and the pH difference between intrapore and inner solutions. In the unmodified membrane, SMX^–^ anions can take part in the formation of hydrogen bonds with both sulfonic acid groups of the polymer (owing to the amino group of the analyte) and hydration shells of counter-ions forming the Debye layer with sulfonic acid groups of the polymer (owing to sulfonamide group). At the same time, the presence of SMX^–^ anions in the membrane pores may lead to some membrane deactivation to cations because of steric factors and the binding of functional groups.

The US treatment of the polymer solution on membrane preparation affected the DP-sensors sensitivity to SMX^–^ insignificantly, but the sensitivity to TMP^+^/TMP increased (Figure 8). It seemed that the more developed system of pores and channels promotes the transfer of bulk cations and molecules of TMP into the membrane. The sensitivity of DP-sensors based on hybrid membranes to both analytes was higher than that based on MF-4SC or MF-4SC-US (Figure 8). It was caused by the formation of hydrophobic centers, which promoted the sorption of the organic ions into the membrane owing to stacking interactions. The highest sensitivity to both analytes was observed for DP-sensors based on MF-4SC/1.0 wt% CNTs-COO^−^ (Figure 8). This may be due to the high availability of CNT surfaces for the analytes in the presence of covalently bound carboxyl groups. Whereas functional groups of the dopant in MF-4SC/CNTs-SO_3_^−^ and MF-4SC/CNTs-NH_3_^+^ membranes were the parts of bulk moieties, which could limit the access of the organic ions to the CNTs surface. The higher sensitivity of DP-sensors to the analytes in the case of MF-4SC/1.0 wt% CNTs-COO^−^ membrane in comparison with MF-4SC/1.0 wt% CNTs-SO_3_^−^ could be due to the more pronounced ability of carboxyl groups to form associates with amino groups of the analytes (Figure 8). The enhanced diffusion permeability of MF-4SC/1.0 wt% CNTs-COO^−^ membrane, compared with that of the unmodified membranes and MF-4SC/1.0 wt% CNTs-SO_3_^−^ membrane, also favored the increase in the analyte concentration in the membrane because of the increasing size of the intrapore space. An increase in the sensitivity of DP-sensors based on MF-4SC/CNTs-NH_3_^+^ to SMX^−^ anions was expected due to the enhanced nonselective transport (established for inorganic ions), as well as the possibility of electrostatic interactions and the formation of hydrogen bonds between sulfonamide moieties of the analyte and surface groups of the dopant. However, the sensitivity of DP-sensors based on MF-4SC/1.0 wt% CNTs-NH_3_^+^ to both analytes was higher than that based on the MF-4SC membrane and lower than on the others (Figure 8). It was possible that (3-aminopropyl)trimethoxysilanol groups on the CNTs-NH_3_^+^ surface were hardly accessible to the organic anions because of their large size. Additionally, the pore cross-linking, due to the formation of hydrogen bonds between the amine-containing groups of the dopant and sulfonic acid groups of the polymer, could cause steric limitations of the organic ions to transfer into the MF-4SC/CNTs-NH_3_^+^ membrane pores.

#### 3.3.2. Analysis of Model Solutions and Pharmaceuticals

A multisensory system based on MF-4SC/1.0 wt% CNTs-SO_3_^−^ (DP-sensor I) and MF-4SC/1.0 wt% CNTs-NH_3_^+^ (DP-sensor II) membranes was chosen for the simultaneous determination of SMX^−^ and TMP^+^/TMP in the solutions with concentrations ranging from 1.0 × 10^−5^ to 1.0 × 10^−3^ M (pH 4.53–8.31). The system of calibration equations for the chosen array of DP-sensors was as in Equation (5). The average scatter of experimental response values of DP-sensors relative to those predicted by the calibration equations was 8 mV, and the variance of the response was no greater than 23 mV^2^.

(5) $$\left\{\begin{array}{c} \Delta \varphi_{D}^{I}=-232-28.7\cdot pSMX+6.2\cdot pTMP+16.98\cdot pH,\\ \Delta \varphi_{D}^{II}=-214-25.4\cdot pSMX+3.4\cdot pTMP+13.6\cdot pH. \end{array}\right.$$

The main characteristics of the SMX^−^ and TMP^+^/TMP determination in the model solutions with different concentration ratios using the DP-sensor array are shown in Table 3. The LODs of SMX^−^ and TMP^+^/TMP were 3.5 × 10^−7^ and 1.3 × 10^−7^, respectively. The relative error of the SMX^−^ determination was 1.4–15% at 4–18% RSD. The relative error of the TMP^+^/TMP determination was 4–12% at 3–18% RSD. The array of DP-sensors based on MF-4SC/1.0 wt% CNTs-SO_3_^−^ and MF-4SC /1.0 wt% CNTs-NH_3_^+^ membranes was used to determine the active ingredients in the Biseptol^®^ 120 mg preparation (tablets). The determined concentrations of SMX^−^ and TMP^+^/TMP in the pharmaceutical solution using the multisensory system were (0.38 ± 0.02) × 10^−4^ and (0.66 ± 0.05) × 10^−5^ M, respectively (Table 4). These values corresponded to the concentrations of SMX and TMP in the preparation of 96 ± 6 and 19.0 ± 1.3 mg, respectively. The relative errors of the SMX and TMP determination as compared with the content declared by the manufacturer were 4% (at 6% RSD) and 5% (at 7% RSD), respectively.

**Figure 9:**
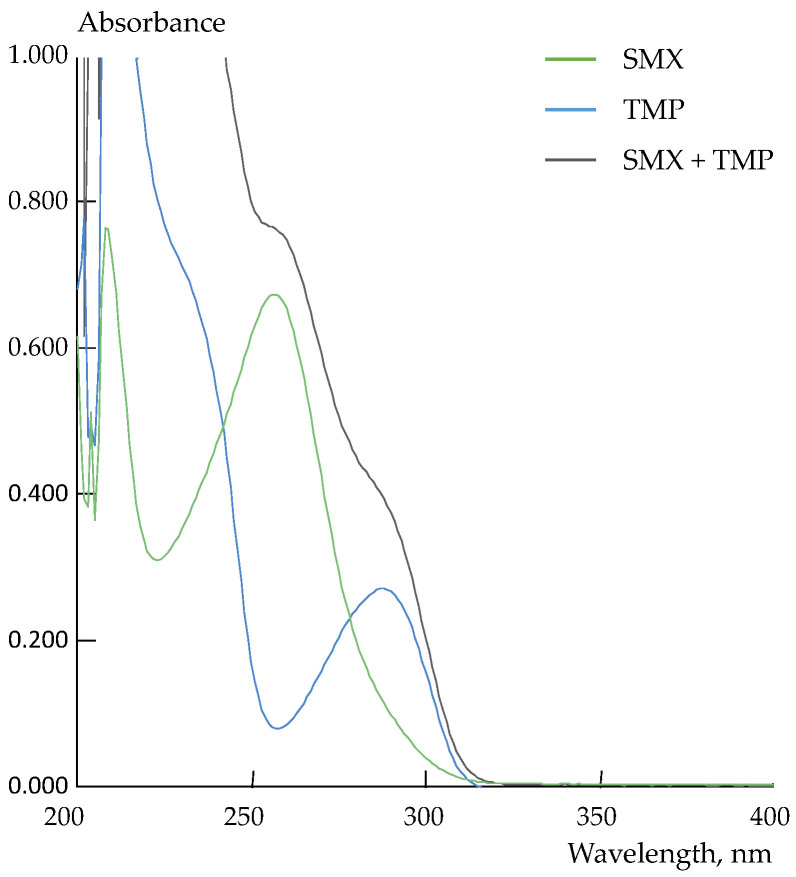
UV-spectra of SMX, TMP, and their equimolar solutions with the concentration of the components of 4.0 × 10^−5^ M at pH 10.0.

Spectrophotometry was used as a reference method for the SMX and TMP determination in the preparation. The best peak separation on the UV-spectra of individual solutions of SMX (λ = 257 nm) and TMP (λ = 287 nm) was observed at pH = 10.0 (Figure 9).

Despite the satisfactory resolution of the characteristic peaks, the difference in the absorbance intensity of the analytes did not allow us to use the traditional approach for their simultaneous determination (Figure 9). Therefore, a system of multidimensional regression equations was obtained to discriminate the responses. The calibration equations were established in solutions containing both analytes at the wavelengths corresponding to absorbance maxima of the individual solutions of the analytes. The system of calibration equations was as in Equation (6). The average scatters of the experimental values of absorbance relative to those predicted by the calibration equations and the variance of absorbance were no greater than 5 × 10^−3^ and 2 × 10^−5^, respectively.

(6) $$\left\{\begin{array}{c} A_{257}=16.8\cdot 10^{3}\cdot c_{SMX}+2.2\cdot 10^{3}\cdot c_{TMP},\\ A_{287}=2.9\cdot 10^{3}\cdot c_{SMX}+6.9\cdot 10^{3}\cdot c_{TMP}. \end{array}\right.$$

The results of the spectrophotometric determination of SMX and TMP in the Biseptol^®^ 120 mg preparation are shown in Table 4. The results of the potentiometric and spectrophotometric analyses were in good agreement. The relative errors of the SMX and TMP determination in the preparation using the array of DP-sensors as compared with the concentration found by spectrophotometry were 1.9 and 2% (Table 4).

#### 3.3.3. Stability and Reproducibility of the Multisensory System Characteristics

The uniform distribution of CNTs in the matrix of perfluorosulfonic acid membranes, as well as hydrophobic interactions between them, prevent dopant leaching from the membranes. The fouling of perfluorosulfonic acid membranes could be possible due to the accumulation of organic analyte ions in their pores, affecting the sorption and transport properties of the membranes [27]. Membranes in the K^+^-form were used to eliminate it in DP-sensors. The high affinity of perfluorosulfonic acid membranes to K^+^ ions provided a fast and complete regeneration of the samples via equilibration with a 0.1 M KCl solution. Moreover, the spatial separation of the membrane boundarieswith the test and reference solutions of DP-sensors and minimized the diffusion of components from the analyzed media into the membrane bulk. The re-estimation of the multisensory system calibration characteristics showed no statistically significant difference after one year of the membrane use if the membrane was stored in deionized water and washed between measurement series (Table 5).

## 4. Conclusions

A potentiometric multisensory system for the simultaneous determination of sulfamethoxazole and trimethoprim in combination drugs was developed. The cross-sensitivity to both analytes was provided by the use of hybrid materials based on MF-4SC perfluorosulfonic acid membranes and functionalized CNTs. The CNT surface was modified by carboxyl, sulfonic acid, or (3-aminopropyl)trimethoxysilanol groups. The functional groups on the CNT surface encouraged their location in the membrane hydrophilic pores. This increased the membrane sorption ability of the analytes owing to their ion exchange and stacking interactions with a dopant. The influence of the CNT concentration and the properties of their surface, as well as preliminary ultrasonic treatment of the polymer and CNT solution before the casting of hybrid membranes, on their ion exchange capacity, water uptake, and transport properties was revealed. An array of cross-sensitive sensors based on the MF-4SC membranes containing 1.0 wt% of CNTs with sulfonic acid or (3-aminopropyl)trimethoxysilanol groups enables to provide the simultaneous determination of sulfamethoxazole and trimethoprim in aqueous solutions with a concentration ranging from 1.0 × 10^−5^ to 1.0 × 10^−3^ M (pH 4.53–8.31). The detection limits of sulfamethoxazole and trimethoprim were 3.5 × 10^−7^ and 1.3 × 10^−7^ M. A good agreement of the results of the potentiometric analysis with both the results of the spectrophotometric analysis as a reference method and the content declared by the manufacturer was observed. The relative errors of sulfamethoxazole and trimethoprim determination using the multisensory system in the combination drug as compared to the content of active ingredients declared by the manufacturer were 4% (at 6% RSD) and 5% (at 7% RSD). It should be noted that the pharmaceutical analysis using multisensory systems did not require probe pretreatment, apart from a little dilution of the preparation. The possibility of long-term use of the sensor array without re-calibration was shown.

## Figures and Tables

**Table 1:** The characteristics of electrochemical sensors for the simultaneous determination of SMX and TMP (the numerator is for SMX, and the denominator is for TMP).

Analysis Object	Method; Sensor Composition	Linear Range, M; LOD, M	Relative Error, %; RSD, %	Comments	Ref.
Natural water	DPV; Paste electrode: paraffin/MWCNTs/SbNPs	(0.1–0.7)×10^−6^/(0.1–0.7)×10^−6^; 2.4×10^−8^/3.1×10^−8^	0.9–4.8/1.6–2.8; -/-	A pH correction to 7.0; polishing and thorough washing before every measurement was required	[5]
Urine model solution	DPV; SPE: polyester/MWCNTs/PBNCs	(1.0–10.0)×10^−6^/(0.1–10.0)×10^−6^; 3.8×10^−8^/6.0×10^−8^	0.6–7.7/1.2–8.7; 3.5–4/2.1–3.3	A pH correction to 7.0; stability up to 3 weeks	[6]
Tap water	DPV; SPE: parchment paper/rGNRs	1.0–10.0)×10^−6^/(1.0–10.0)×10^−6^; 0.9×10^−7^/0.4×10^−7^	1.51–2.17/0.14–0.48; 1.06–3.54/1.96–3.81	A pH correction to 6.0	[7]
Tap water	DPV; GCE/ GR-ZnO nanorods	1.0×10^−6^–2.2×10^−4^/1.0×10^−6^–1.8×10^−4^; 0.4×10^−6^/0.3×10^−6^	3.8–3/4.7–1.0; -/-	A pH correction to 7.0; stability up to 3 weeks	[8]
Lake water			0.9–5/2.3–6.5; -/-		
Urine			1.2–6/2.3–5.2; -/-		
Serum			3.5–5.4/3.2–4.5; -/-		
Tablets	SWV; GCE	5.5×10^−5^–3.95×10^−4^/1.05×10^−5^–1.04×10^−4^; 8.52×10^−6^/9.31×10^−7^	0.53–2.41/1.89–3.20; -/-	A pH correction to 6.0; rapid deactivation of the surface due to adsorption of oxidation and reduction products	[9]
Oral suspension			0.46–7.35/0–4.05; -/-		
Injections			0.97–1.88/−1.00–2.10; -/-		
Bactrim^®^ tablets	DPV; Diamond electrode dopped with boron	(0.39–3.2)×10^−5^/(0.69–5.5)×10^−6^; 0.651×10^−7^/0.630×10^−7^	−9.4/–4.5; -/-	A pH correction to 7.0	[10]
Bactrim F^®^ tablets			−2.7/–3.5; -/-		
Aquaculture waters	Potentiometry; PVC-membrane with MIP	5×10^−5^–1×10^−3^/1×10^−6^–5×10^−5^; 1.7×10^−5^/3.0×10^−7^	0.4–5.4/0.3–3.1; -/-	A pH correction to 5.0; the sensor worked in flow injection mode	[17]

RSD—relative standard deviation; DPV—differential pulse voltammetry; SWV—squarewave voltammetry; SPE—screen-printed electrode; GCE—glass carbon electrode; MWCNTs—multiwall carbon nanotubes; SbNPs—antimony nanoparticles; PBNCs—Prussian blue nanocubes; rGNRs—reduced graphene nanoribbons; PVC-membrane—polyvinyl chloride membrane.

**Table 2:** The ionic conductivity (σ, mS/cm) in contact with water at 30 °C and diffusion permeability (P, cm^2^/s) in 0.02–0.5 M KCl solution of the membranes in the K^+^-form (the relative errors in the estimation of conductivity and diffusion permeability are below 10 and 1.0%).

Membrane	Dopant Concentration, wt%	σ, mS/cm	P·10^8^, cm^2^/s
MF-4SC	-	3.8	7.94
MF-4SC-US	-	5.7	15.6
MF-4SC/CNTs-COO^−^	0.5	4.3	13.9
	1.0	4.7	16.8
	1.5	4.5	15.3
MF-4SC/CNTs-SO_3_^−^	0.5	4.6	9.83
	1.0	5.2	12.6
	1.5	6.8	18.0
MF-4SC/CNTs-NH_3_^+^	0.5	5.0	48.9
	1.0	5.1	56.5
	1.5	4.7	28.5

**Table 3:** The characteristics of the SMX^−^ and TMP^+^/TMP determination in the model solutions (*c* = 1.0 × 10^−5^–1.0 × 10^−3^ M, pH 4.53–8.31) using the array of DP-sensors based on MF-4SC/1.0 wt% CNTs-SO_3_^−^ and MF-4SC/1.0 wt% CNTs-NH_3_^+^ membranes.

Analyte	LOD, M	Relative Error, %	RSD,% (n = 4, *p* = 0.95)
SMX^−^	3.5 × 10^−7^	1.4–15	4–18
TMP^+^/TMP	1.3 × 10^−7^	4–12	3–18

**Table 4:** The analysis of the Biseptol^®^ 120 mg (Adamed Pharma, Czosnów, Poland) using the spectrophotometric technique and the DP-sensor system based on MF-4SC/1.0 wt% CNTs-SO_3_^−^ and MF-4SC/1.0 wt% CNTs-NH_3_^+^ membranes.

Method		The DP-Sensor Array	Spectrophotometry
c, M (pharmaceutical solution)	SMX^−^	(0.38 ± 0.02) × 10^−4^	(0.387 ± 0.007) × 10^−4^
	TMP^+^/TMP	(0.66 ± 0.05) × 10^−5^	(0.671 ± 0.012) × 10^−5^
c, mg (preparation)	SMX	96 ± 6	98.1 ± 1.7
	TMP	19.0 ± 1.3	19.5 ± 0.3
RSD, % (n = 6, *p* = 0.95)	SMX	6	2
	TMP	7	2
Relative error *, %	SMX	4	1.9
	TMP	5	3

* The relative error was calculated compared with the content declared by the manufacturer.

**Table 5:** Comparison of calibration characteristics of the developed multisensory system before and after using it for a year.

Membrane	b_0_, mV		b_1_, mV/pSMX		b_2_, mV/pTMP		b_3_, mV/pH		*t*-Test, f = 11, *p* = 0.95	*F*-Test, f_1_ = 4, f_2_ = 7, *p* = 0.95
	t	F	t	F	t	F	t	F		
MF-4SC/1.0 wt% CNTs-SO_3_^−^	1.03	0.82	0.91	0.84	0.91	0.74	0.96	0.87	2.20	4.12
MF-4SC/1.0 wt% CNTs-NH_3_^+^	1.95	1.04	0.55	1.01	0.95	0.97	1.40	1.09		

## Data Availability

Not applicable.
